# Hyperkalemic Recurrent Bilateral Lower Extremity Weakness in a Patient on Hemodialysis

**DOI:** 10.1155/2012/243501

**Published:** 2012-05-13

**Authors:** Getaw Worku Hassen, Suzanne Newstead, Lorraine Maria Giordano

**Affiliations:** Department of Emergency Medicine, Metropolitan Hospital Center, NYMC, 1901 First Avenue, New York, NY 10029, USA

## Abstract

Hyperkalemia is a severe life-threatening electrolyte disorder that commonly affects the cardiac conductivity and contractility. Ascending paralysis affecting the extremities with focal neurological deficit as well as quadriparesis and a seizure associated with hyperkalemia has been reported in the literature. Here, we describe a case of isolated recurrent lower extremity paralysis and an episode of seizure in an 83-year-old patient with end-stage renal disease on hemodialysis.

## 1. Introduction

Hyperkalemia is a well-known electrolyte disorder especially in patients with end-stage renal disease [1]. This clinical entity may be associated with electrocardiogram (ECG) changes that could lead to life-threatening arrhythmic disorders such as ventricular fibrillation and asystole [2–5]. Changes in the electrolytes, especially changes, in the potassium level, leading to ECG changes and paralysis can vary as a result of drugs, diet, metabolic changes and failure to follow up regular dialysis [6–8]. Electrolyte disturbances can also lead to seizure [5]. The level of serum potassium does not always correlate with the ECG changes or degree of paralysis. The disorders associated with hyperkalemia depend on a number of factors such as the rapidity of the development of hyperkalemia and the presence of other electrolyte disorders and the status of the kidney function. The aim of this case report is to highlight and raise awareness for uncommon noncardiac presentation of hyperkalemia.

## 2. Case Report

Here, we report a case of an 83-year-old Hispanic male with end-stage renal disease (ESRD) on dialysis three times a week who presented to the emergency department with the chief complaint of bilateral lower extremity weakness. The patient noted the weakness in his leg after having his early dinner around 3PM. He first noticed weakness in his knee joint and later he had trouble getting out of his chair and walking to the restroom. Initially he managed to walk with help. Over the next several hours the weakness in his legs persisted and became so severe that he decided to come to the hospital.

The patient stated that he had similar problems 3 days prior to presentation and on the day of his dialysis and the symptoms resolved after a 3-hour dialysis. Prior to these events and in between the two episodes the patient had normal, age appropriate activities of daily living. He walked without help and without any difficulty.

His past medical history included was significant for end-stage renal disease on hemodialysis three times a week, hypertension, and coronary artery disease with stent placement, myocardial infarction, and seizure disorder.

The patient had undergone a colectomy for perforated bowel with colostomy several years ago.

The patient denied cough, fever, diarrhea, and change in bowel or urinary habits. There was no prior history of trauma.

His home medication list included Aspirin 81 mg per day, Clopidogrel 75 mg per day, Simvastatin 40 mg per day, Metoprolol 25 mg per day, Multivitamin one tablet a day, folic acid one tablet twice a day, Calcium acetate 667 mg two tablet with each meal daily, Phenytoin 100 mg twice a day, and Omeprazol 20 mg per day.

The review of systems was significant for a recent history of new-onset seizure that required intubation for air way protection. Patient was admitted to Medical Intensive Care Unit (MICU). Patient was later discharged home on Phenytoin. Shortly before this admission, patient has presented to the hospital multiple times with serum potassium levels as high as 8.3 milliEquivalents/liter (mEq/L).

Physical examination was remarkable for bilateral lower extremity weakness pronounced at the hips (motor strength 2/5 in both legs). Furthermore, cogwheel rigidity was appreciated bilaterally, more pronounced in the knee joints. Sensory system examination and the reflexes were within normal limit. No saddle anesthesia was appreciated on neurological examination. Motor strength and cranial nerve examination were essentially unremarkable.

**Figure 1:**
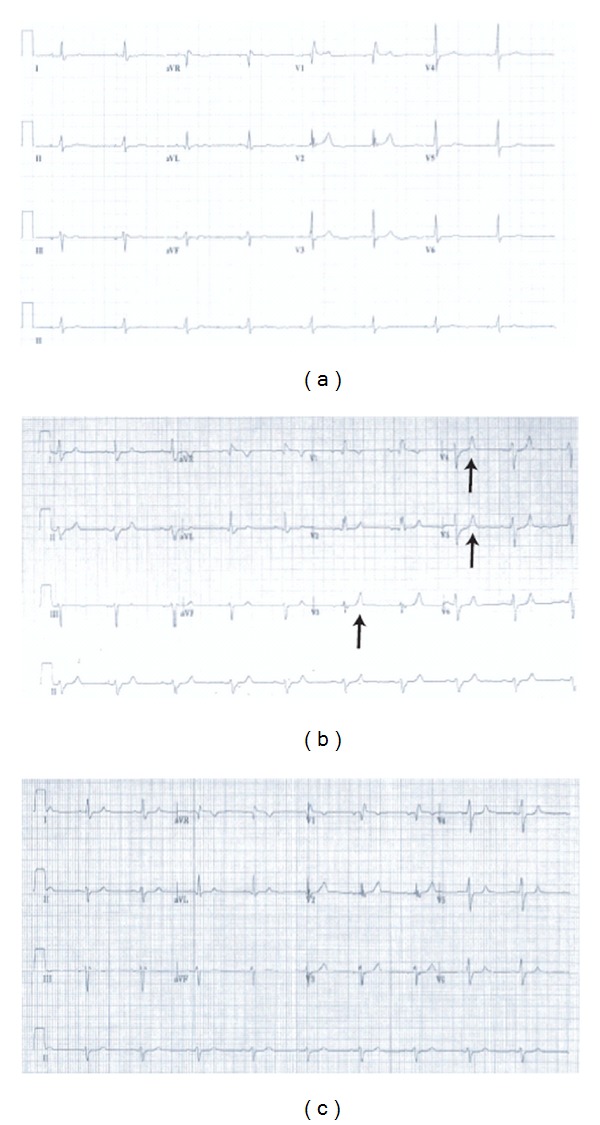
ECG changes during the hyperkalemic event, after treatment and a comparative old ECG. (a) Old ECG from March 2007; (b) peaked T-waves (arrows) in the setting of acute hyperkalemia; (c) improved EKG after treatment.

ECG revealed peaked T-wave and the serum chemistry study was remarkable for potassium of 7.8 mEq/dL (Figure 1(b)).

A portable chest radiograph showed no acute infiltration or fluid overload.

The patient's diet includes rice, beans, pork chops, beef, chicken, and salad.

In the emergency department, the patient was given 10 cc of Calcium gluconate, 5 units of regular insulin, 25 cc of Dextrose 50%, and 60 mg of oral Sodium polystyrene sulfonate immediately based on the ECG and laboratory abnormalities.

Patient was attached to the monitor and observed with serial neurological exam, ECG and serial labs.

After an hour, patient's weakness and cogwheel rigidity partially improved and the patient was able to stand with help. The patient was transferred to the MICU service for close monitored observation. Patient was subsequently dialyzed. Repeat laboratory values and the ECG returned to previous values (Figures 1(a) and 1(c)), and the patient was able to stand up and walk without difficulty maintaining his preevent status.

## 3. Discussion

Ascending paralysis is a rare complication of hyperkalemia [6, 7, 9–11]. This type of paralysis resembles the one that is observed in Guillain-Barre Syndrome [12, 13]. It has been reported that this entity has been observed in patients with end-stage renal disease on hemodialysis or it has been precipitated as a result of antibiotic therapy [7] or in the context of drug use and abuse [6, 8].

Hyperkalemic paralysis can occur as an inherited disease such as familial hyperkalemic periodic paralysis (HYPP) [14] or in a form of secondary hyperkalemia paralysis [15] as a result of excessive potassium intake; drug effects that inhibit the secretion of potassium or decrease the excretion of potassium in the kidney [7] increased potassium production as in the case of rhabdomyolysis or abnormal potassium distribution [6].

Hyperkalemic paralysis is diagnosed after ruling out other causes for the paralysis and improvement of the paralysis after correcting the abnormal serum potassium level. There was no apparent cause for the paralysis in this patient and the paralysis improved after the initial medical therapy partially and complete recovery was achieved after the subsequent dialysis.

Apparent cause for hyperkalemia was not noted from the medication list or from the patient's diet.

Reviewing the literate, so far single episodes of paralysis were reported [9]. In our case the patient had recurrent paralysis of the lower extremities which was associated with high serum potassium and improvement of the system after correcting the potassium level.

In addition, this patient had a new-onset seizure in the recent past which required intubation for airway protection. This seizure activity could have been the result of hyperkalemia. It is well known that electrolyte disturbance, particularly hyperkalemia, can cause seizure [5]. Multiple factors can contribute to the development of seizure including electrolyte disorders. We believe that the new-onset seizure in this patient occurred as a result of electrolyte disorder, especially hyperkalemia.

Ascending paralysis is a rare but possible complication of hyperkalemia in a hemodialysis patient [9, 11]. Here must be a high index of suspicion for hyperkalemia in the setting of paralysis in a hemodialysis patient, especially after other causes of paralysis, such as stroke, have been ruled out. Obtaining an early ECG is crucial to diagnose a life-threatening, potentially lethal hyperkalemia. Hyperkalemic ascending paralysis can affect muscles of the respiratory system and progress to ventilatory failure. This is relevant in the use of succinylcholine for rapid-sequence induction for intubation (RSI) could be fatal as a result of increased serum potassium that may occur [8].

## 4. Conclusion

 Cardiac-related abnormalities secondary to hyperkalemia are common sources of emergency presentation. Noncardiac complications of hyperkalemia such as paralysis and seizure are uncommon; however, these conditions should be part of the differential of hyperkalemia for patients with ESRD. Urgent recognition and appropriate early therapy are essential.
